# Myocardial infarction in a population-based cohort of patients with biopsy-confirmed giant cell arteritis in southern Sweden

**DOI:** 10.1136/rmdopen-2023-003960

**Published:** 2024-04-10

**Authors:** Pavlos Stamatis, Moman Aladdin Mohammad, Karl Gisslander, Peter A Merkel, Martin Englund, Carl Turesson, David Erlinge, Aladdin J Mohammad

**Affiliations:** 1 Department of Clinical Sciences Lund, Rheumatology, Lund University, Lund, Sweden; 2 Department of Rheumatology, Sunderby Hospital, Luleå, Sweden; 3 Department of Clinical Sciences Lund, Cardiology, Lund University, Lund, Sweden; 4 Division of Rheumatology, Department of Medicine, and Division of Epidemiology, Department of Biostatistics, Epidemiology, and Informatics, University of Pennsylvania, Philadelphia, PA, USA; 5 Department of Clinical Sciences Lund, Orthopedics, Clinical Epidemiology Unit, Lund University, Lund, Sweden; 6 Department of Clinical Sciences Malmö, Rheumatology, Lund Universtiy, Malmö, Sweden; 7 Department of Medicine, University of Cambridge, Cambridge, UK

**Keywords:** Giant Cell Arteritis, Vasculitis, Cardiovascular Diseases, Incidence, Mortality

## Abstract

**Objectives:**

To determine the incidence rate (IR) of myocardial infarction (MI), relative risk of MI, and impact of incident MI on mortality in individuals with biopsy-confirmed giant cell arteritis (GCA).

**Methods:**

MIs in individuals diagnosed with GCA 1998–2016 in Skåne, Sweden were identified by searching the SWEDEHEART register, a record of all patients receiving care for MI in a coronary care unit (CCU). The regional diagnosis database, with subsequent case review, identified GCA patients receiving care for MI outside of a CCU. A cohort of 10 reference subjects for each GCA case, matched for age, sex and area of residence, was used to calculate the incidence rate ratio (IRR) of MI in GCA to that in the general population.

**Results:**

The GCA cohort comprised 1134 individuals. During 7958 person-years of follow-up, 102 were diagnosed with incident MI, yielding an IR of 12.8 per 1000 person-years (95% CI 10.3 to 15.3). The IR was highest in the 30 days following GCA diagnosis and declined thereafter. The IRR of MI in GCA to that of the background population was 1.29 (95% CI 1.05 to 1.59). Mortality was higher in GCA patients who experienced incident MI than in those without MI (HR 2.8; 95% CI 2.2 to 3.6).

**Conclusions:**

The highest incidence of MI occurs within the 30 days following diagnosis of GCA. Individuals with GCA have a moderately increased risk of MI compared with a reference population. Incident MI has a major impact on mortality in GCA.

**WHAT IS ALREADY KNOWN ON THIS TOPIC**

- Patients with giant cell arteritis (GCA) suffer elevated rates of cardiovascular morbidities compared with the general population. Mortality in GCA is slightly increased in the first year postdiagnosis, mainly due to cardiovascular disease.

**WHAT THIS STUDY ADDS**

- Incidence of acute myocardial infarction (MI) is highest during the 30 days following GCA diagnosis. GCA patients experience a greater rate of MI versus a demographically matched cohort from the general population. Mortality is higher in individuals diagnosed with GCA who experience incident MI than in those without MI.

**HOW THIS STUDY MIGHT AFFECT RESEARCH, PRACTICE OR POLICY**

- Research is needed to address the question of a time-limited prophylactic treatment for MI early in the course of GCA.

## Introduction

Giant cell arteritis (GCA) is the most common vasculitis among elderly persons, with incidence peaking after age 70.^1 2^ The ratio of females to males in populations from North Europe and North America is nearly 3:1.^1 3–6^ GCA affects predominantly large-sized and medium-sized extracranial arteries.^7^ Depending on the intensity of the inflammatory response and the distribution of affected arteries, presenting symptoms may be headache, vision changes, scalp tenderness, jaw or arm claudication, and other vascular-related manifestations of disease.^8–10^ Most patients experience constitutional symptoms including arthralgias, malaise, fever and weight loss.^8 9^ Elevated inflammatory markers, anaemia of inflammation and thrombocytosis are common laboratory findings at diagnosis.^8 11^ Stroke and ischaemia affecting vision are the most feared manifestations of the disease.^12 13^ Glucocorticoids (GC) remain the mainstay of treatment,^14 15^ and interleukin 6 blockade and methotrexate have emerged as an effective glucocorticoid-sparing treatment.^15 16^ Antiplatelet therapy is not routinely used in the treatment of GCA.^15 16^

Cardiovascular disease (CVD) is the leading cause of mortality in Sweden and other western countries.^17 18^ Similar to GCA, the incidence of myocardial infarction (MI) increases with age and peaks in those older than 70 years.^19^ GCA presents potential risk factors for CVD, including inflammation of the circulatory system and the detrimental effects of GC therapy. The long-term use of GCs is associated with weight gain, diabetes mellitus and hypertension, all known risk factors for CVD.^20^ Results of recent trials indicate a potential role of anti-inflammatory drugs in the treatment or prevention of CVD; subjects receiving anti-inflammatory drugs (canakinumab, colchicine) exhibited better cardiovascular outcomes than comparison groups.^21 22^

We conducted a population-based study in southern Sweden using a large cohort of patients with temporal artery biopsy (TAB)-confirmed GCA and two comprehensive national and regional registers, aiming to (i) determine the incidence rate (IR) of MI in patients with biopsy-confirmed GCA, (ii) compare the IR of MI in GCA to that in a matched reference population without vasculitis and (iii) quantify the impact of incident MI on mortality in GCA.

## Methods

### Study area

The study was conducted in Skåne, the southernmost region of Sweden. Skåne had a population of 1 324 565 in December 2016 (13.3% of the total Swedish population in 2.7% of the area),^23^ more 95% of which was Caucasian. The Skåne region includes both urban and rural areas. The healthcare system in Sweden is largely tax-funded and ensures access to services for the entire population.

### GCA cohort

The case identification and retrieval of patients with biopsy-confirmed GCA in this study have been described in detail.^24^ All patients who underwent TAB 1998–2016 were identified from the Department of Pathology in Skåne, which operates at the major hospitals in the area: Skåne University Hospital in Lund and Malmö, Helsingborg Hospital and the Central Hospital in Kristianstad. All pathology reports were reviewed. Patients were identified as having GCA if the report stated a diagnosis of GCA, temporal arteritis, granulomatous arteritis, or unequivocally indicated infiltration of mononuclear cells into the arterial wall, with or without giant cells.

### Case identification and ascertainment of MIs

#### SWEDEHEART register

The SWEDEHEART register contains data of all patients admitted to coronary care units (CCUs) undergoing care for acute coronary disease.^25^ Data of demographics, risk factors, medical history, medical treatment prior to admission, electrocardiographic changes, biochemical markers, other clinical features and investigations, medical treatment in hospital, interventions, hospital outcome, discharge diagnoses, and medications at discharge are recorded.^25^ The register captures ~75% of all non-ST-elevation MI and >95% of all ST-elevation MIs in Sweden.^25^

#### Skåne Healthcare Register

The Skåne Healthcare Register (SHR) is a comprehensive administrative register initiated in 1998 that contains information regarding healthcare services in the region of Skåne, Sweden. Visits to physicians, nurses and other healthcare professionals are catalogued by a unique personal identity number. Diagnoses are reported in the SHR using the International Classification of Diseases, 10th revision (ICD-10). In addition to diagnostic codes, the SHR contains information that constitutes the basis of monetary reimbursement: sex, age, place of residence, date of visit and information regarding the healthcare provider.^26^

#### Case identification and ascertainment of MIs

Using personal identification numbers all persons with GCA experiencing MI and receiving care at any CCU were identified through the SWEDEHEART register. Information on type of MI, date of admission to CCU and date of discharge were collected. Patients diagnosed with MI in medical departments outside of a CCU, and hence not listed in the SWEDEHEART register, were identified through the SHR using all ICD-10 codes indicating MI assigned after admission to any hospital in the area. Cases of MI events identified by diagnostic codes were reviewed to verify diagnosis according to the fourth universal definition of MI.^27^

#### Estimation of incidence rate ratio of MI (GCA vs reference population)

**Figure 1:**
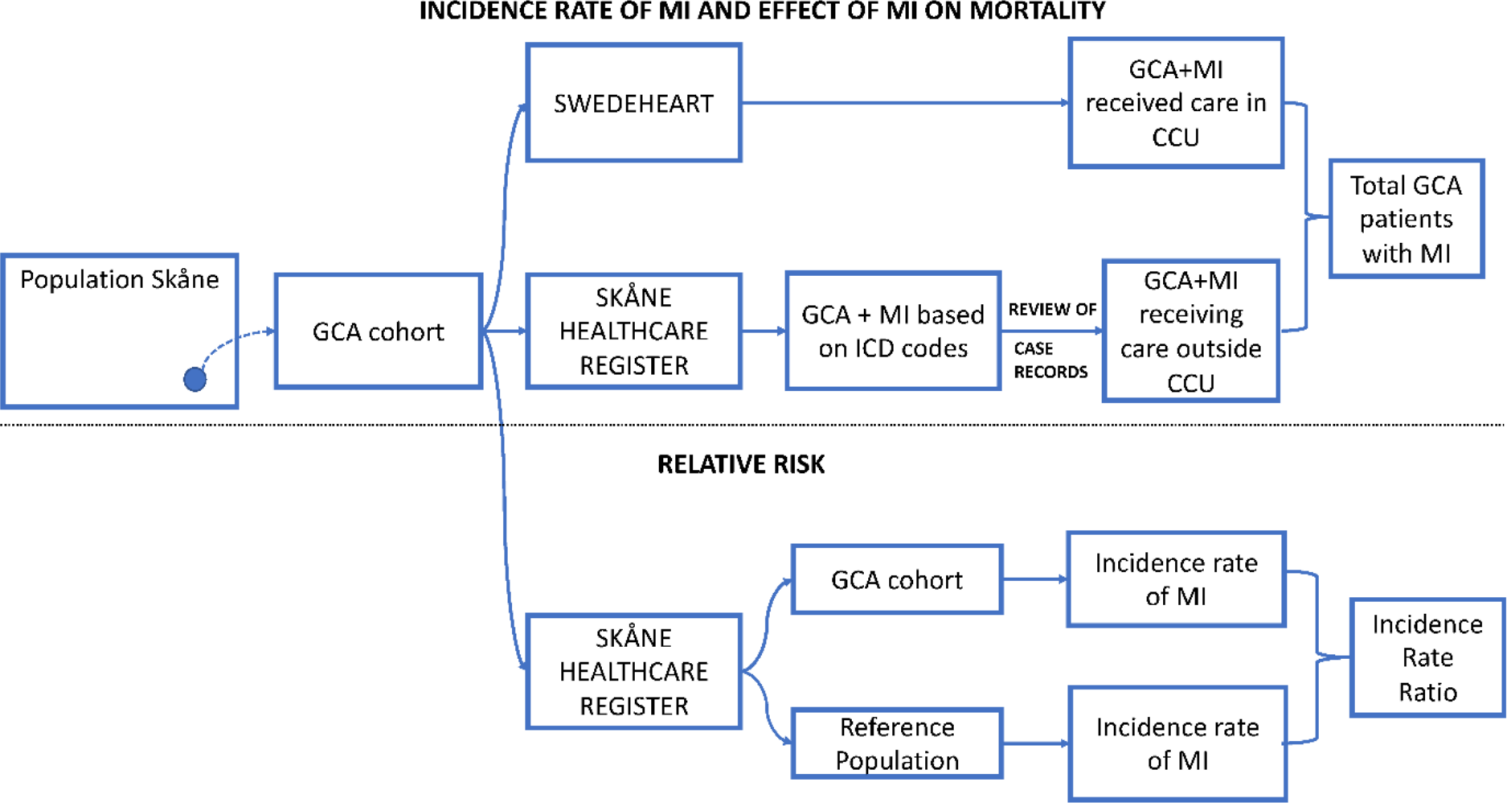
Schematic of case identification and ascertainment of myocardial infarction in patients with giant cell arteritis and a reference population. CCU, Coranary Care Unit; GCA, Giant Cell Arteritis; MI, Myocardial Infarction.

To calculate the incident rate ratio (IRR) of MI in individuals diagnosed with GCA to that in the general population, for each case of GCA, 10 reference subjects matched for age, sex and area of residence were randomly selected through the SHR. Since we lacked approval to review case records of reference subjects, MI was identified based solely on assigned ICD-10 codes, assuming 100% accuracy. To control for possible diagnostic bias, in the IRR calculation the MI estimate of the GCA cohort also relied only on ICD-10 codes, without review of case records (figure 1).

### Statistical analysis

To estimate the IR of MI, the numerator was the number of MI cases and the denominator was the sum of patient follow-up years. For GCA, follow-up time was calculated from the date of diagnosis. The person-years of follow-up was calculated from date of GCA diagnosis/index date to the first MI event, the date of death or end of study (1 April 2018). To calculate IR of MI relative to time after GCA diagnosis, the person-years of follow-up were divided into defined periods: 0–30 days, 0–3 months, 4–6 months, 7–9 months, 10–12 months, 0–12 months, 13–24 months, 25–60 months, 61–120 months and 121 months to end of follow-up.

To compare the IR of MI in persons with GCA with that of the reference population, we calculated the IRR with 95% CIs. An index date corresponding to the GCA diagnosis date was applied to each patient’s 10 matched reference subjects. To reduce the risk of misclassifying a prevalent MI as incident, since the SHR was established in 1998, we included only patients with diagnosis/index date of 2000 and later. We calculated the IRR using a cut-off that included MI events occurring only after the GCA diagnosis/index date. In a sensitivity analysis, we also included events that occurred within the 30 days prior to GCA diagnosis.

We investigated the relationship between incident MI and mortality in patients with GCA using a time-dependent Cox regression model. Subjects were followed from GCA diagnosis to the end of the study. Diagnosis of MI after diagnosis of GCA was considered exposure (time-dependent variable), and the outcome of interest was death. The relationship between the outcome and other variables/predictors such as sex, prior MI and age at the time of GCA diagnosis was assessed in univariate and multivariate analyses.

## Results

### Incidence of MI in individuals with GCA

**Figure 2:**
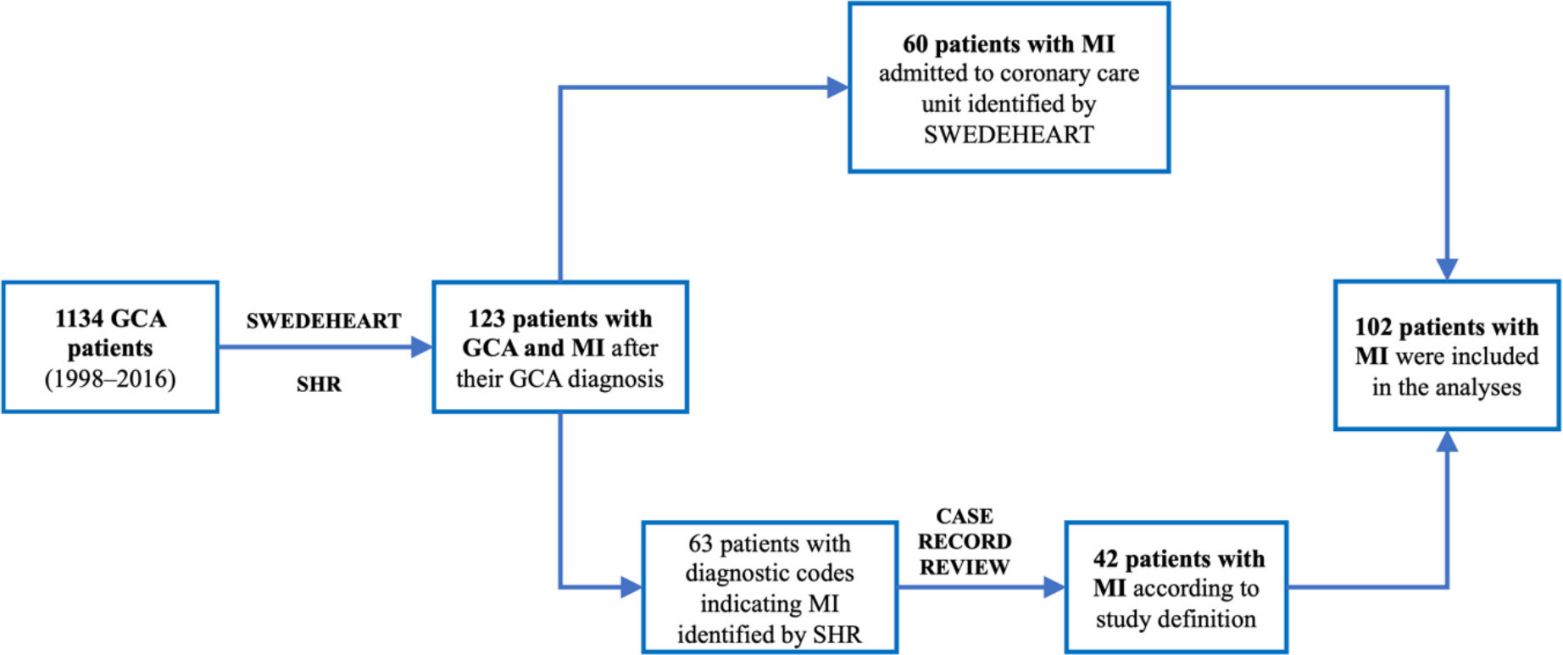
Flowchart illustrating the number of patients with biopsy-confirmed giant cell arteritis who suffered myocardial infarction after diagnosis of giant cell arteritis, identified via the SWEDEHEART register and the Skåne Healthcare Register. GCA, giant cell arteritis; MI, myocardial infarction; SHR, Skåne Healthcare Register.

The GCA cohort comprised 1134 individuals diagnosed with biopsy-confirmed GCA (813 females, 72%). The SWEDEHEART register identified 60 cases of MI in the cohort. Through the SHR, 63 additional persons were identified with an ICD code indicating acute MI (figure 2), confirmed by medical record review in 42. Hence, 102 patients (68 females) who suffered an incident MI after diagnosis of GCA (table 1) were included in the analyses for the calculation of MI IR and in the time-dependent Cox regression model.

**Table 1:** Characteristics of patients with giant cell arteritis (GCA) with and without myocardial infarction (MI) after diagnosis of giant cell arteritis in a population-based cohort from southern Sweden

	With MI n=102	Without MI n=1032
Female sex, n (%)	68 (66.7)	745 (72.2)
Mean age at diagnosis of GCA; years (SD)	77.1 (8.1)	75 (8.0)
Mean age at MI diagnosis; years (SD)	81.8 (7.7)	NA
Mean follow-up time; years (SD)	8.3 (5.3)	7.2 (4.8)
Past history of MI, n (%)	12 (11.8)	35 (3.4)
All-cause mortality, n (%)	74 (72.5)	465 (45.1)
Mean age at death; years (SD)	86.6 (7.0)	85.1 (7.1)

NA, not applicable.

**Figure 3:**
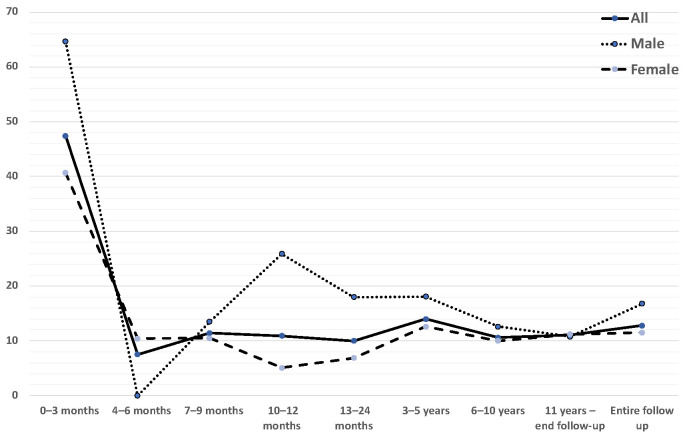
Overall and sex-specific incidence rates of myocardial infarction per 1000 person-year in patients with biopsy-confirmed giant cell arteritis during the follow-up period.

The 102 of 1134 GCA patients suffering at least one MI yielded a cumulative incidence of 9% (95% CI 7.2 to 10.7). Among patients receiving care in a CCU, the cumulative incidence was 5.3% (95% CI 4 to 6.6) (online supplemental figure 1). During a follow-up time of 7957.5 person-years, the IR of MI in patients with biopsy-confirmed GCA was 12.8 per 1000 person-years (95% CI 10.3 to 15.3) for all patients, 11.5 (95% CI 8.7 to 14.2) in females, and 16.8 (95% CI 11.2 to 22.5) in males. The rate was highest in the 30 days following GCA diagnosis (figure 3). The MI rate was higher in males than in females, except in the 30 days post-GCA (60.4 per 1000 person-years in females vs 38.2 in males). When we excluded from the analysis patients with a history of prior MI (12 patients, 6 women and 6 men), the IR of MI in patients with biopsy-confirmed GCA was 11.6 per 1000 person-years (95% CI 9.2 to 14) for all patients, 10.6 (95% CI 7.9 to 13.6) in females and 14.7 (95% CI 9.3 to 20.2) in males.

10.1136/rmdopen-2023-003960.supp1 Supplementary data

Overall and sex-specific MI IRs for discrete time periods after diagnosis of GCA are presented in table 2 as well as overall and sex-specific IRs for the entire study period.

**Table 2:** Incidence rates* and sex-specific incidence rates* of myocardial infarction (MI) in patients with biopsy-confirmed giant cell arteritis (GCA), in Skåne, Sweden

	All			Men			Women		
**Time period**	**MI**	**PY**	**IR (95% CI**)	**MI**	**PY**	**IR (95% CI**)	**MI**	**PY**	**IR (95% CI**)
**First month post-GCA diagnosis**									
0–1 month	5	92.4	54.1 (6.7 to 101.5)	1	26.17	38.2 (0 to 113.1)	4	66.27	60.4 (1.2 to 119.5)
**First year post-GCA diagnosis**									
0–3 months	13	274.4	47.4 (21.6 to 73.1)	5	77.4	64.6 (8 to 121.2)	8	197.1	40.6 (12.5 to 68.7)
4–6 months	2	268.28	7.5 (0 to 17.8)	0	75.4	0	2	192.9	10.4 (0 to 24.7)
7–9 months	3	263.8	11.4 (0 to 24.2)	1	73.9	13.5 (0 to 40.1)	2	189.9	10.5 (0 to 25.1)
10–12 months	3	274.7	10.9 (0 to 23.3)	2	77.2	25.9 (0 to 61.8)	1	197.5	5.1 (0 to 15)
0–12 months	21	1081.2	19.4 (11.1 to 27.7)	8	303.9	26.3 (8.1 to 44.6)	13	777.4	16.7 (7.6 to 25.8)
**Year 2 to end of follow-up**									
13–24 months	10	1004.1	10 (3.8 to 16.1)	5	277.6	18 (2.2 to 33.8)	5	726.5	6.9 (0.8 to 12.9)
Years 3–5	34	2428.8	14 (9.3 to 18.7)	11	606.8	18.1 (7.4 to 28.8)	23	1822	12.6 (7.5 to 17.8)
Years 6–10	25	2361	10.6 (6.4 to 14.7)	7	553.9	12.6 (3.3 to 22)	18	1807.2	10 (5.4 to 14.6)
Years 11 to end of follow-up	12	1082.5	11.1 (4.8 to 17.4)	3	278.9	10.8 (0 to 22.9)	9	803.6	11.2 (3.9 to 18.5)
**Entire follow-up period**									
Entire follow-up	102	7957.5	12.8 (10.3 to 15.3)	34	2021	16.8 (11.2 to 22.5)	68	5936.5	11.5 (8.7 to 14.2)

*Per 1000 PY.

IR, incidence rate; PY, person-years.

### MI IR in GCA versus reference population

The analysis included 1013 patients (719 females) diagnosed with GCA from 2000 through 2016 along with 10 127 (7189 females) reference subjects identified through the SHR. Linking the GCA cohort to the SHR revealed 99 (68 females) with an ICD-code of MI assigned subsequent to GCA diagnosis. The corresponding number for the reference population was 777 (529 females). The IRR was 1.29 (95% CI 1.05 to 1.59) in the main analysis, considering only MI that occurred after the diagnosis/index date. The IRR including MI in the 30 days prior to GCA diagnosis/index date, in the sensitivity analysis, was 1.30 (95% CI 1.06 to 1.60). Sex-specific IR and IRR are presented in table 3 along with results of sensitivity analysis.

**Table 3:** The relative risk of myocardial infarction (MI), presented as incidence rate ratio, in patients with biopsy-confirmed giant cell arteritis compared with a reference population

	Cases			Reference			Rate ratio	
	n	Person-years	Rate	n	Person-years	Rate	Rate ratio	95% CI
All	99	6685.1	14.8	777	67 673.4	11.5	1.29	1.05 to 1.59
Female	68	4942.8	13.8	529	51 057.1	10.4	1.33	1.03 to 1.71
Male	31	1742.4	17.8	248	16 616.3	14.9	1.19	0.82 to 1.73
**Sensitivity analysis including MI in 30 days preceding index date**								
All	101	6683.5	15.1	787	67 712.3	11.6	1.30	1.06 to 1.60
Female	69	4942.5	13.9	537	51 086.4	10.5	1.32	1.03 to 1.71
Male	32	1741.0	18.3	250	16 625.9	15.0	1.22	0.85 to 1.77

n, number of MI.

### Impact of incident MI on survival in patients with GCA

The time-dependent Cox regression model showed incidence of MI after diagnosis of GCA to be associated with an increased risk of death with an HR of 2.81 (95% CI 2.17 to 3.63). This association remained in adjusted models (table 4). HRs for death after incident MI were higher in males (4.29; 95% CI 2.70 to 6.81) than in females (2.32; 95% CI 1.7 to 3.17). Myocardial infarct at any time prior to GCA diagnosis was also associated with increased mortality (table 4).

**Table 4:** Univariate and multivariate-adjusted HRs with 95% CIs of risk of death among patients with giant cell arteritis (GCA) and myocardial infarction (MI) in southern Sweden

		Univariate analysis	Multivariate analyses		
**Predictors**	**Patients (n)**	**Unadjusted HR**	Model 1*	**Model 2†**	**Model 3‡**
MI after diagnosis of GCA§	102	2.81 (2.17 to 3.63)	2.71 (2.01 to 3.34)	2.71 (2.09 to 3.52)	2.07 (1.59 to 2.69)
MI prior to diagnosis of GCA	47	1.88 (1.25 to 2.83)	1.55 (1.02 to 2.34)	1.53 (1 to 2.34)	1.29 (0.85 to 1.96)
Female sex	813	0.95 (0.79 to 1.15)	NI	0.98 (0.81 to 1.19)	0.89 (0.73 to 1.08)
Age at diagnosis of GCA	1134	1.11 (1.1 to 1.13)	NI	NI	1.11 (1.09 to 1.12)

*Adjusted for previous MI.

†Adjusted for previous MI and sex.

‡Adjusted for previous MI, sex and age.

§Time dependent covariate.

NI, not included.

## Discussion

We conducted epidemiological assessment of the incidence and outcome of MI in patients with biopsy-confirmed GCA using data of a large population-based cohort from a well-defined area in southern Sweden. The IR of MI in patients with GCA was 12.8/1000 person-years, with the highest rate in the 30 days following GCA diagnosis. Patients with GCA showed a moderately increased risk for MI compared with reference subjects from the general population. Individuals with GCA suffering from an MI had a 2.8-fold rate of death compared with those without MI.

The IR of MI in this study is comparable to those reported in European studies,^28 29^ but our estimate is slightly higher than reported in a matched cohort study derived from The Health Improvement Network (UK) database that calculated MI IR as 10 per 1000 person-years.^29^ Differences in study design and MI case ascertainment (based only on diagnostic codes in the UK-based study) may explain the discrepancy. Recent research in France found lower cumulative incidence of MI in patients with biopsy-confirmed GCA: 5.2%.^28^ That study included only GCA patients admitted to cardiology intensive care units with incident MI. The cumulative incidence of the French study is almost identical to the cumulative incidence of our study if we had included only patients identified via SWEDEHEART (5.3%), that is, only those who were admitted to CCUs in Sweden.

Our research confirms the results of previous studies indicating an increased incidence of MI during the months immediately following diagnosis of GCA.^28 29^ The pathogenic mechanism of this phenomenon is not known. The cited study from France showed that patients with GCA most commonly experience type II MI, without angiography findings of acute atherothrombotic plaque disruption.^28^ The strong systemic inflammatory response and the detrimental effects of GC therapy may increase the demand for oxygen in the heart muscle, leading to an imbalance of oxygen demand and supply. Sepsis, anaemia and hypoxia are known triggers for type II MI.^30^ In addition, the persistent low-grade inflammatory response commonly present in patients with GCA may accelerate the rate of atherosclerosis and increase the long-term risk for type I MI.^31 32^ Recently, a high rate of thromboembolic events and stroke in the 3 months postdiagnosis of ANCA-associated vasculitis was reported in patients from the same area in Sweden.^33 34^ Similarly, Wegener’s Clinical Occurrence of Thrombosis study reported a median time from enrolment to venous thrombotic event of 2.1 months.^35^ These findings support the hypothesis of systemic inflammation possibly playing a role in the development of a cardiovascular event in vasculitis.

The IR of MI in patients with GCA was higher in males than in females except for the 30 days post-GCA. As mentioned, the French study found the majority of the GCA-related MIs to be type II (four of five), and a GCA flare was the only triggering factor identified in three of the four.^28^ A larger proportion of females experience type II MI compared with type 1, and female sex has been identified as a risk factor for type II MI.^36 37^ In 183 subjects in our cohort undergoing TAB, the mean erythrocyte sedimentation rate at the time of diagnosis was 81±26.6 mm/hour, and the median C reactive protein level was 99 (IQR 56–143) mg/L.^38^ A possible explanation for the female bias in the IR of MI in the 30 days post-GCA diagnosis is that the combination of increased inflammatory response and secondary inflammatory anaemia results in an imbalance in the oxygen supply and demand in the myocardium, leading a type II MI unrelated to acute coronary atherothrombosis.^8 11^

There is limited, low-grade evidence supporting antiplatelet therapy in patients with GCA, and its routine use for GCA management was not recommended by the EULAR 2018 task force for large vessel vasculitis.^15^ The ACR 2021 GCA guidelines recommend the use of antiplatelet therapy only in patients with critical or flow-limiting involvement of vertebral or carotid arteries.^16^ Research has primarily investigated the effect of antiplatelet therapy on ischaemic complications in general (stroke, visual manifestations, MI) and not solely on MI.^39^ Our findings of an increased rate of MI in the initial months following diagnosis of GCA may prompt a discussion of the clinical utility of time-limited prophylactic treatment in patients newly diagnosed with GCA.

The incidence of MI was higher in individuals with biopsy-confirmed GCA compared with the reference population. The increased incidence of MI in patients with GCA, especially early in the disease course conflicts with observations of lower body mass index, lower plasma glucose levels and lower lipid levels reported in health survey participants who subsequently developed GCA.^40–42^ In our sensitivity analysis, we examined the IR and IRR, taking into account MI events that occurred within the 30 days leading up to the diagnosis of GCA. This consideration was based on the median diagnostic delay of GCA in our cohort, previously shown to be 24 days (IQR 9–45).^43^ The results of the IRR remained nearly unchanged after incorporating this timeframe. These findings suggest that the increased risk of incident MI in patients with early GCA may be attributed to a combination of systemic and vascular inflammation, as well as the treatment with high doses of GCs, rather than solely to the pre-existing risk profile before the onset of GCA.

Individuals with prior MI and those with MI after GCA diagnosis exhibited, respectively, a 1.9-fold and 2.8-fold risk of death compared with GCA patients without MI. Mortality rate after an incident MI was greater in males than in females in our study. The findings are in agreement with two meta-analyses and several observational studies demonstrating excess mortality in GCA, primarily due to CVD.^44–48^ In our study, incident MI predicted higher mortality after adjustment for sex, age at GCA diagnosis, and history of MI prior to the onset of GCA.

The strengths of our study include the large sample size and the population-based setting, enabling the identification of all patients with biopsy-confirmed GCA over a 19-year period. Additionally, the well-validated SWEDEHEART register and the SHR allowed identification of nearly all individuals who had interacted with a healthcare provider in our region during the study period. For inpatient care from 1998 through 2017, close to 100% of consultations were assigned a diagnostic code in the SHR.^26^ The multi-source identification of MI in our study, the centralised nature of the healthcare in Skåne, and the subsequent review of case records afforded highly accurate determination of the IRs. The study provides a time frame for risk of MI in patients with newly diagnosed biopsy-confirmed GCA in the near, mid-term and long-term.

The study has some limitations. We investigated risk of MI in patients with biopsy-confirmed GCA, and results may not be generalisable to patients with biopsy-negative GCA, including isolated large-vessel GCA, or with polymyalgia rheumatica with subclinical vasculitis. It is possible that patients with GCA with a negative TAB, including some patients with predominantly aortic or branch arterial disease, have different rate of MI from patients with a positive TAB. Although every effort was made to include the majority of individuals suffering from an MI, we cannot exclude the possibility of undiagnosed MI in persons who died before reaching a healthcare facility. Our study also lacks data on important CVD risk factors such as smoking, hypertension, diabetes and hyperlipidaemia.

## Conclusions

The risk of MI is highest in the 30 days following diagnosis of GCA and subsides after 6 months. Individuals with biopsy-confirmed GCA have a moderately increased risk for MI compared with the demographically matched general population. Persons diagnosed with GCA and incident MI show a risk of death 2.8 times that of those with GCA and no MI. Further studies should investigate the impact of inflammation, GC treatment, antiplatelet therapy, treatment with targeted biological drugs and traditional risk factors on the risk of MI in individuals with biopsy-confirmed GCA.

## Data Availability

No data are available. Raw data are protected by confidentiality laws in Sweden and cannot be shared. All data relevant to the study are included in the article. For further information contact the corresponding author.
