# Supplementary material for: Myocardial infarction in a population-based cohort of patients with biopsy-confirmed giant cell arteritis in southern Sweden
Source: RMD Open. 2024 Apr 10;10(2):e003960. doi: 10.1136/rmdopen-2023-003960 (PMC11015192; doi:10.1136/rmdopen-2023-003960)

Supplementary Figure 1 Absolute numbers and proportions of patients with giant cell arteritis (GCA) suffering myocardial infarction (MI) in coronary care units (CCU) and outside CCU, in a cohort of 1134 patients with biopsy-confirmed GCA

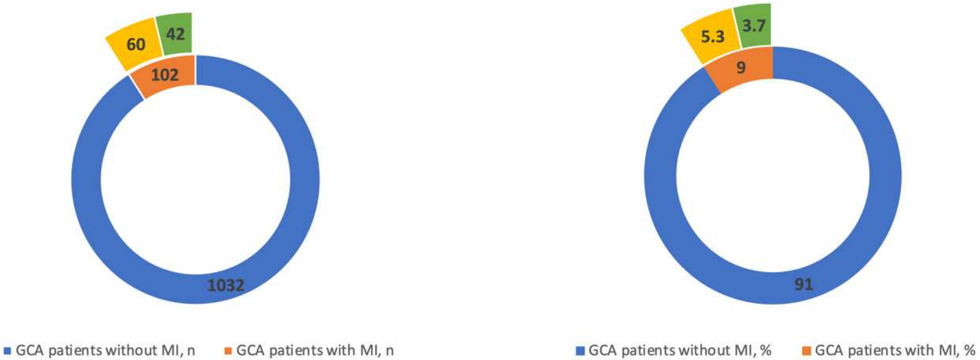

Supplement: Supplementary data [file rmdopen-2023-003960supp001.pdf]
